# Expectations of a new opt‐out system of consent for deceased organ donation in England: A qualitative interview study

**DOI:** 10.1111/hex.13394

**Published:** 2021-12-24

**Authors:** Pippa K. Bailey, Hannah Lyons, Fergus J. Caskey, Yoav Ben‐Shlomo, Mohammed Al‐Talib, Adarsh Babu, Lucy E. Selman

**Affiliations:** ^1^ Department of Population Health Sciences, Bristol Medical School University of Bristol Bristol UK; ^2^ Department of Renal Medicine, Southmead Hospital North Bristol NHS Trust Bristol UK; ^3^ Leeds School of Medicine University of Leeds Leeds UK; ^4^ Department of Renal Medicine, Gloucestershire Royal Hospital Gloucestershire Hospitals NHS Foundation Trust Gloucester UK

**Keywords:** deceased donation, opt‐out law, organ donation, qualitative interviews, transplantation

## Abstract

**Introduction:**

In 2020 England moved to an opt‐out deceased donation law. We aimed to investigate the views of a mixed stakeholder group comprising people with kidney disease, family members and healthcare practitioners towards the change in legislation. We investigated the expected impacts of the new legislation on deceased‐donor and living‐donor transplantation, and views on media campaigns regarding the law change.

**Methods:**

We undertook in‐depth qualitative interviews with people with kidney disease (*n* = 13), their family members (*n* = 4) and healthcare practitioners (*n* = 15). Purposive sampling was used to ensure diversity for patients and healthcare practitioners. Family members were recruited through snowball sampling and posters. Interviews were audio‐recorded and transcribed verbatim. Transcripts were analysed using thematic analysis.

**Results:**

Three themes with six subthemes were identified: (i) Expectations of impact (Hopeful patients; Cautious healthcare professionals), (ii) Living‐donor transplantation (Divergent views; Unchanged clinical recommendations), (iii) Media campaigns (Single message; Highlighting recipient benefits). Patients expected the law change would result in more deceased‐donor transplant opportunities.

**Conclusions:**

Clinicians should ensure patients and families are aware of the current evidence regarding the impact of opt‐out consent: expectations of an increased likelihood of receiving a deceased‐donor transplant are not currently supported by the evidence. This may help to prevent a decline in living‐donor transplantation seen in other countries with similar legislation. Media campaigns should include a focus on the impact of organ receipt.

**Patient or Public Contribution:**

Two patient representatives from the Kidney Disease Health Integration Team, Primrose Granville and Soumeya Bouacida, contributed to the content and design of the study documents.

## 1 INTRODUCTION

The number of individuals waiting for an organ transplant in the United Kingdom is greater than the number of organs available for transplant.^1^ Before 2015, all the countries of the United Kingdom operated under an ‘opt‐in’ system in which individuals who wished to donate their organs after death actively registered on the organ donor register. In 2015, Wales became the first country in the United Kingdom to introduce ‘opt‐out’ legislation. In this system, individuals who do not wish to donate their organs after death register this by ‘opting‐out’. Individuals who have not ‘opted‐out’ are presumed to consent to donation. Opt‐out legislation was implemented in England in May 2020, in Scotland in March 2021 and a public consultation is underway in Northern Ireland.

A recent comparison of organ donation and transplantation rates in 35 countries found no strong evidence of a difference between opt‐in and opt‐out countries.^2^ However, countries with opt‐out legislation had fewer living organ donors per million population (pmp) than those with opt‐in legislation (4.8 vs. 15.7 pmp).^2^ Most living‐donor transplants are kidney transplants, with a minority liver, lung and intestinal. In general, outcomes for living‐donor transplants are better than for deceased‐donor transplants with respect to both transplant and patient survival.^1^, ^3^, ^4^, ^5^, ^6^, ^7^, ^8^, ^9^, ^10^ Living organ donation is associated with risks to the donor, but these are small. Perioperative mortality is estimated to be 0.01%–0.03%^11^, ^12^, ^13^ in living kidney donation and 0.2% in living liver donation.^14^, ^15^ Survival at 11 years is comparable for liver donors, kidney donors and matched nondonors (mortality at 11 years 1.2%, 1.2% and 1.4%, respectively).^15^

The reasons behind the observed association between opt‐out legislation and lower rates of living donation are not well understood. In the context of an opt‐out system, potential donors, transplant candidates and healthcare practitioners may perceive living donation (and the accompanying risks) as unacceptable or unnecessary if they anticipate an increased likelihood of receiving a deceased‐donor organ.^16^

The aim of this qualitative interview study was to investigate the views of people with kidney disease, family members and healthcare practitioners on the change to opt‐out legislation in England. We aimed to investigate (i) the expected impacts of the change in legislation, including with respect to living donation and living‐donor transplantation, (ii) whether the expected impacts of the change in legislation would affect decision‐making regarding living‐donation and transplantation, and (iii) views on the focus of media campaigns accompanying the change in the law. Findings will help to ensure healthcare interactions and media campaigns address the expectations and concerns of these relevant stakeholders. Understanding the impact of the legislation on patients and healthcare professionals' transplant decision‐making may allow intervention to prevent a reduction in living‐donor transplants that has been observed in other countries.

## 2 MATERIALS AND METHODS

The study was undertaken at two English hospitals (a transplanting centre and a transplant referring centre). Semi‐structured qualitative interviews were undertaken with English‐speaking adults aged ≥18 years and <75 years, from the following groups:

1. People with advanced kidney disease (chronic kidney disease stages 4 and 5, and those receiving kidney replacement therapy).
2. Family members of people with advanced kidney disease including living donors.
3. Healthcare practitioners who work in nephrology and transplantation.

Individuals who lacked the mental capacity to consent to participation were not included.

Patients were invited to participate by post, in person and through posters in outpatient clinics and haemodialysis units. Purposive sampling of patient participants was undertaken to ensure diversity in sex, age, ethnicity, kidney disease history and socioeconomic status. Family members were recruited via hospital posters and by ‘snowball sampling’ through participants with kidney disease. Healthcare practitioners were invited to participate by Chief Investigator (P. K. B.) via email, and were purposively sampled to ensure diversity in sex, age, ethnicity and clinical role.

Interviews were undertaken by P. K. B., an academic nephrologist with formal training and experience in qualitative research methodology. She was known to healthcare practitioners but was not known to any of the patients or family participants. Patient and family participants were told in the study information sheet that P. K. B. is a kidney doctor and researcher working at the University of Bristol. This study was undertaken as part of another larger study: the topic guides are provided (Supporting Information Material—example topic guides). Participants were asked about their expectations of the impact of the change in legislation on donation rates, and whether their expectations would change their behaviours. Interviews were either undertaken over the telephone or face‐to‐face at a location of the participant's choosing. Written consent for participation was provided at the time of face‐to‐face interviews. For telephone interviews, verbal consent was recorded and written consent was confirmed via post. Participant demographic data were collected at the time of the interview. A £20 voucher for participation was given to all participants.

Interviews were audio‐recorded, transcribed verbatim, and analysed using inductive thematic analysis,^17^ as described by Braun and Clarke.^18^ Anonymized transcripts were uploaded to NVivo software^19^ for analysis. All transcripts were coded by P. K. B. and a subset inductively and independently analysed by two other researchers (M. A.‐T. an Academic Clinical Fellow in renal medicine, and H. L., an MRes student in Health Sciences Research). Codes and any discrepancies were discussed to maximize rigour, reliability and reflexivity. Data collection and analysis were conducted concurrently, employing an iterative approach. The sample size was determined by reaching theoretical theme saturation when few or no new relevant data were identified in successive interviews.^20^ Saturation was assessed with respect to the sample of mixed stakeholders, not to subsamples within. The report was written with reference to the consolidated criteria for reporting qualitative studies (COREQ).^21^ The study received Health Research Authority and NHS Research Ethics Committee approval (Reference 19/WM/0320).

## 3 RESULTS

Thirty‐three of Ninety‐two (36%) invited individuals agreed to participate (Table 1) but one individual was then unavailable for an interview. Interviews ranged from 13 to 74 min, with a mean duration of 42 min.

**Table 1:** Participant characteristics

Characteristics	*n* = 32
	Number (%)
Sex	
Female	17 (53)
Male	15 (47)
Age group (years)	
20–39	4 (13)
40–59	23 (72)
60–79	5 (16)
Ethnicity	
White	27 (84)
Other ethnic groups (Asian/Asian British; Black/African/Caribbean/Black British; mixed/multiple ethnic groups; other ethnic groups)^a^	5 (16)
Marital status	
Single	9 (28)
Married/long‐term partner	20 (63)
Other (divorced; widowed/bereaved)	3 (9)
Participant group	
People with advanced kidney disease^a^	13 (41)
Family members^b^	4 (13)
Healthcare practitioners Transplant nurses or coordinators Home dialysis nurses Nurses other, e.g., ward, haemodialysis Transplant physicians/surgeons	15 (47) 5 3 4 3
People with kidney disease and family—the highest level of education	*n* = 17
Secondary school	1 (6)^c^
Vocational/technical training	7 (41)^c^
University undergraduate degree	2 (12)^c^
University postgraduate degree	4 (24)^c^
Not disclosed	3 (18)^c^
People with kidney disease and family—employment status	*n* = 17
Unemployed	8 (47)^c^
Full or part‐time employment	5 (29)^c^
Retired and other (e.g., student, homemaker)^a^	4 (24)^c^

^a^ Unable to provide information on subgroups due to small numbers in groups risking identification.

^b^ One family member was also a healthcare practitioner. They are included here as a family member.

^c^ % of 17 subgroup sample not % of 32 total sample.

Three themes with six subthemes were identified: (i) Expectations of impact (Hopeful patients; Cautious healthcare professionals), (ii) Living‐donor transplantation (Divergent views; Unchanged clinical recommendations), (iii) Media campaigns (Single message; Highlighting recipient benefits) (Table 2). Quotes are followed by participant identification (ID) number and participant group.

**Table 2:** Themes and illustrative quotes

Theme	Subtheme	Illustrative quotes
Expectations of impact	Hopeful patients	‘I mean fortunately things are changing and now it's got to the point where it's an opt‐out situation’. (ID18/Patient) ‘I think the change in law that's going to end up with default donation of pieces of body on death is a great move’. (ID12/Patient) ‘Interviewer: Do you think it's going to change your chances of getting a kidney from somebody who's died? Participant: Logically, yes, it should do’. (ID23/Patient) ‘I think if somebody's not happy to donate anything he will say that…so better is the law which has assumed you have agreed…that will be better because, for example a lot of people want to do something but they are too lazy to! I know by me, because sometimes I think “Oh that it is worth to do that, but maybe tomorrow”. And tomorrow I can die!’ (ID28/Patient) ‘Yes I mean I thought that they were doing the pilot scheme where they were having an opt in rather than opt out and I thought that was a great idea…And the thing is like, you'll probably find then that we're a lot more likely to get kidneys for the rarer types of patients and another thing I've noticed is like, ethnic minorities tend to struggle to get donations because of the size of their communities and things like religion and things that stop them from. So I mean this would just improve chances for people like that as well’. (ID30/Patient)
	Cautious healthcare professionals	‘I don't think there is going to be a sudden rush of kidneys because people are going to be saying “Woohoo, they haven't opted out therefore we can whip their kidneys out”’. (ID07/Transplant nurse or coordinator) ‘…everybody still knows that if at the end the family says no, it says no…I don't really think it is going to make any difference’. (ID20/Transplant nurse or coordinator) ‘The quality of the kidneys are not necessarily going to be all good either, so…I don't anticipate that I'll change my practice when it's on the opt‐out legislation, and the likely 10‐15% increase [in donations]’. (ID26/Transplant physician or surgeon) ‘I think the [English] population thinks that the waiting list is going to disappear overnight, however…[Welsh colleagues/friends] are sort of saying “It's not going to be the huge game changer that everyone thinks”’. (ID10/Transplant nurse or coordinator) ‘On the news it said that Wales took years to see any change and I think that most people, especially people invested in this, people wanting a transplant, know about things like that, so I don't really think it is going to make any difference’. (ID20/Transplant nurse or coordinator) ‘I think people are not understanding that the change in law is going to help much … because it's just like dying at the right time isn't it? Which not many people do, so how much of a difference, maybe one percent difference kind of thing? That's where these people have false hope, isn't it? So, I think we probably need to educate people a bit more that this is actually not going to bring much change’ (ID22/Transplant nurse or coordinator)
Living‐donor transplantation	Divergent views	‘…hopefully it will open up the conversation about transplantation within families, because of that and it might just the whole “What would you do if I needed a kidney?” conversation will be more transparent so people that do need an LKD [living kidney donor], they have already had those sort of conversations’. (ID05/Transplant nurse or coordinator) ‘I think who's gonna donate? It's gonna be probably family or very close friend and they're doing it for altruistic reasons of love, care and whatever for the person who's been affected’. (ID06/Patient) ‘Interviewer: Do you think it is going to have an impact on living donation when we go to an opt‐out deceased system? Participant: No, because I think the majority of people have read what has come on Facebook and everybody still knows that if at the end the family says no, it says no’. (ID20/Transplant nurse or coordinator) ‘Participant: Since the law, since 2011 with enhanced organ utilisation procurement, live donor numbers have gone down and people now think that they can sit on a dialysis machine and the organ will come to them. Interviewer: And you think that's going to get worse? Participant: Yes, it will get worse. It will get worse and the quality of, I mean you can play the data however you like, but the organs that we are going to get are not going to be good quality organs because good quality people don't die of conditions that we can then use kidneys from’. (ID19/Transplant physician or surgeon)
	Unchanged clinical recommendations	‘I wouldn't say “Don't have a living donor because actually there is going to be hundreds of kidneys” because my personal opinion is I don't think there will be’. (ID07/Transplant nurse or coordinator) ‘I think it remains equally, if not more important, to still have all those conversations and educate people that it's just because the organ donation opt‐out thing is changing next year doesn't mean to say, actually, you're any more likely to get a transplant’. (ID01/Nurse, other e.g., ward, haemodialysis) ‘I don't think you need to dwell on it but I think what you need to do is say obviously the opt out legislation that's coming in next Spring may slightly increase your chances of having a kidney from the transplant waiting list, however the majority of people that die in and out of hospital, are not eligible to be donors because of the very specific criteria that they have to fulfil so it's unlikely to affect your waiting time that much. And then follow up with, if you have a live donor option, you know that's [best]’. (ID10/Transplant nurse or coordinator) ‘I don't think even with the opt‐out we'll ever meet the incident patients joining the list. So, yes, in that sense we'll still be delivering the living donor transplantation … I don't anticipate that I'll change my practice when it's on the opt‐out legislation…’ (ID26/Transplant physician or surgeon) ‘In terms of longevity of the kidney transplant, obviously living donor option is the best option…so I think we should still be promoting that, and that shouldn't be changed because of the opt‐out system’. (ID29/Transplant physician or surgeon) ‘The organs that we are going to get are not going to be good quality organs because good quality people don't die of conditions that we can then use kidneys from ….I think if anything the conversation with my patients has got more towards live donation because I think [of] the quality of [deceased] organs that we're putting in’. (ID19/Transplant physician or surgeon)
Media campaign	Single message	‘Do you want to dilute your primary message? Because some of these people have got two things to think about and actually you want them really only to focus on the one…I would say just focus on your primary message “Get Brexit done, get Brexit done, get Brexit done”’. (ID06/Patient) ‘I think it's probably slight information overload for people, when there's already going to be a big change to the way that things are done in the UK. I can see why you'd want to pair them because there's going to be a big publicity campaign anyway, but I think it's probably a bit confusing for people. I think it should be promoted more, nationally more widely, but maybe not at the same time’. (ID29/Transplant physician or surgeon) ‘I don't know, sometimes too much information is too much, so perhaps two campaigns maybe’. (ID31/Family member) ‘I think it [living donation] should be promoted more, nationally more widely, but maybe not at the same time [as the opt‐out law change]’. (ID29/Transplant physician or surgeon) ‘I'm guessing the opt‐out legislation tag will be for all organs, so I think trying to tag‐on kidneys in the main, possibly will make the message too complex’. (ID26/Transplant physician or surgeon)
	Highlighting recipient benefits	‘…some sort of series of adverts that flags up the value of having been given a donated heart, lung or a kidney or a piece of liver lobe or whatever you know…we don't have any public celebration of the successes’. (ID12/Patient) ‘…the message you need to deliver is, like you've got the ability to give someone a second chance at life’. (ID30/Patient) ‘I know there's a big list of (famous) people who have had transplants so I mean, you know … what you need to do is like have some sort of slogan or something for people that say “I did it” Do you know what I mean? I did it sort of thing and you need to like make it a really short and simple message. You know like I've overcome it or whatever. I got over it’. (ID30/Patient)

### 3.1 Expectations of impact: Hopeful patients

All but one patient participant welcomed the change in the law to an opt‐out system, believing that it would result in an increase in organ donations:

I think the change in law that's going to end up with default donation of pieces of body on death is a great move. (ID12/Patient)

Interviewer: Do you think it's going to change your chances of getting a kidney from somebody who's died?

Participant: Logically, yes, it should do. (ID23/Patient)

Two patient participants explained how the change in legislation was more consistent with individual preferences. They thought that people who wanted to donate often failed to act to confirm this, whereas those who objected to donation were more likely to act to avoid donation:

I think if somebody's not happy to donate anything he will say that…so better is the law which has assumed you have agreed…that will be better because, for example a lot of people want to do something but they are too lazy to! I know by me, because sometimes I think ‘Oh that it is worth to do that, but maybe tomorrow’. And tomorrow I can die! (ID28/Patient)

Only one patient had low expectations regarding the law change. He did not believe that the law change would have any impact on donation rates as family members still had to approve donation:

…this is a conversation that I had with the doctor when I was in there, even if they've opted in, it doesn't mean that they necessarily can take those organs, they still have to have permission, they do. So this opt‐in and opt‐out, really, why are they doing it? Because, at the end of the day, you've still got to ask, you've still got to get that permission. (ID32/Patient)

### 3.2 Expectations of impact: Cautious healthcare professionals

The only patient who felt the change in the law would have no impact attributed this view to a conversation with a healthcare professional. In keeping with this, all healthcare professionals interviewed had low expectations regarding the impact of the law change on deceased‐donor numbers. Most anticipated no increase in donor numbers due to the law not changing the practices of healthcare professionals at the time of organ donation, with family members still able to decline donation on an individual's behalf:

…everybody still knows that if at the end the family says no, it says no…I don't really think it is going to make any difference. (ID20/Transplant nurse or coordinator)

Only one healthcare professional anticipated an increase in donor numbers but anticipated that many of the organs made available for transplant would be of poor quality:

The quality of the kidneys are not necessarily going to be all good either, so…I don't anticipate that I'll change my practice when it's on the opt‐out legislation, and the likely 10‐15% increase [in donations]. (ID26/Transplant physician or surgeon)

Two participants (one patient and one healthcare professional) explained that the expectations of the general population should be managed regarding both the potential impact and the time it might take to see change:

I think the [English] population thinks that the waiting list is going to disappear overnight, however…[Welsh colleagues/friends] are sort of saying ‘It's not going to be the huge game changer that everyone thinks’. (ID10/Transplant nurse or coordinator)

So there is I think a change going on but again, like, changes sometimes takes a couple of decades to get through so we're at early stages really now…there is a mindset change going on but I still think we're in quite early days here in Great Britain. (ID18/Patient)

### 3.3 Living‐donor transplantation: Divergent views

Participants expressed different, conflicting views on the anticipated effect on living‐donor transplantation. There was no evidence that views differed according to whether respondents were patients, family members or healthcare professionals. Some participants anticipated no change in living‐donor transplants, others expected rates to decline, and one participant anticipated a positive effect on living donation. This participant described how they believed the change to the opt‐out law and accompanying publicity would trigger conversations between transplant candidates and potential living donors:

…hopefully it will open up the conversation about transplantation within families, because of that and it might just the whole ‘What would you do if I needed a kidney?’ conversation will be more transparent so people that do need an LKD [living kidney donor], they have already had those sort of conversations. (ID05/Transplant nurse or coordinator)

Several participants suggested that the opt‐out law would not change living‐donor transplant activity as it would not change the motivations of living kidney donors:

I think who's gonna donate? It's gonna be probably family or very close friends and they're doing it for altruistic reasons of love, care and whatever for the person who's been affected. (ID06/Patient)

However, in contrast to this view, two participants anticipated a negative impact on living‐donor transplantation. One healthcare professional suggested that living‐donor transplant numbers had already fallen due to previous changes in organ utilisation and anticipated that the change to an opt‐out law would cause a further drop:

Participant: Since the law, since 2011 with enhanced organ utilisation procurement, live donor numbers have gone down and people now think that they can sit on a dialysis machine and the organ will come to them.

Interviewer: And you think that's going to get worse?

Participant: Yes, it will get worse. (ID19/Transplant physician or surgeon)

### 3.4 Living‐donor transplantation: Unchanged clinical recommendations

None of the healthcare professionals interviewed said that the law change would change their clinical practice with respect to discussing living‐donor kidney transplantation, related in part to their low expectations of an increase in deceased donations:

I wouldn't say ‘Don't have a living donor because actually there is going to be hundreds of kidneys’ because my personal opinion is I don't think there will be. (ID07/Transplant nurse or coordinator)

One healthcare professional suggested professionals should continue to ‘promote’ living‐donor transplantation:

In terms of longevity of the kidney transplant, obviously living donor option is the best option…so I think we should still be promoting that, and that shouldn't be changed because of the opt‐out system. (ID29/Transplant physician or surgeon)

### 3.5 Media campaign: Single message

Most participants felt that the media campaign to raise awareness of the change in the law should focus on deceased donation only. Participants felt that adding information about the possibility of living donation was problematic, at risk of generating ‘information overload’ and confusing or diluting the message regarding opt‐out:

Do you want to dilute your primary message? Because some of these people have got two things to think about and actually you want them really only to focus on the one…I would say just focus on your primary message ‘Get Brexit done, get Brexit done, get Brexit done’. (ID06/Patient)

One healthcare professional highlighted that not all organs that can be donated after death can be donated during life, and therefore the message would become too complex. Some healthcare professionals did want to see the active promotion of living donation in the media, although not simultaneous with the opt‐out campaign:

I think it [living donation] should be promoted more, nationally more widely, but maybe not at the same time [as the opt‐out law change]. (ID29/Transplant physician or surgeon)

### 3.6 Media campaign: Highlighting recipient benefits

Several patient participants stated that public awareness and media campaigns associated with the opt‐out law should focus not just on donation but also on the positive impact of receiving an organ transplant. No healthcare professionals suggested this focus.

…some sort of series of adverts that flags up the value of having been given a donated heart, lung or a kidney or a piece of liver lobe or whatever you know…we don't have any public celebration of the successes. (ID12/Patient)

…the message you need to deliver is, like you've got the ability to give someone a second chance at life. (ID30/Patient)

One patient described how sharing her personal need for a transplant with people had increased their interest in deceased donation, suggesting that if such stories were similarly shared as part of a media campaign, they might have a similar effect.

## 4 DISCUSSION

To our knowledge, this is the first qualitative study to describe patient, family and healthcare practitioners' expectations of opt‐out legislation. We found a wide range of expectations regarding the impact on living‐donor transplantation, suggesting collective uncertainty regarding the impact. Healthcare practitioners reported that they would not change their recommendations in light of the change in legislation: it would be worth investigating whether clinical practice mirrors reported intent.

There was a mismatch between the expectations of patients and healthcare professionals: Patient participants were more enthusiastic about the change in the law and expected to see increased donation rates. Evidence regarding the effect of the opt‐out legislation in Wales is mixed. In the 2019–2020 NHS Blood and Transplant (NHSBT) activity report, deceased donation consent rates were comparable for England and Wales (unadjusted overall consent rate 68% vs. 69%).^1^ Between 2016 and 2019 there was evidence of a steady upward trend in the proportion of families consenting to donation after brain death in Wales when compared to England, but this did not continue into 2019–2020, when consent rates in Wales fell compared to previous years.^1^, ^22^ There was no change in consent rates to donation after circulatory death.^1^, ^22^ It may be that this evidence has moderated the expectations of healthcare professionals in England regarding the impact of the change in the law. Many healthcare professionals expressed the belief that deceased organ donation rates would not increase with a change to opt‐out consent as the practices of healthcare professionals at the time of organ donation remain unchanged: family members are still able to overrule an individual's registered decision. This is often described as a ‘soft opt‐out’ system as compared to a ‘hard opt‐out’ system in which families are not involved in decision‐making.^23^ Previous research examining practice in 54 nations has highlighted that in most countries the next‐of‐kin are involved in the decision regarding deceased organ donation regardless of whether the system operates under an opt‐out or opt‐in law or whether the wishes of the deceased to be a donor were known.^24^ While ‘opt‐in’ and ‘opt‐out’ may technically describe the law in a country, it is an oversimplification and forced binary categorisation of varied practice, and may explain why a comparison of organ donation and transplantation rates in 35 countries found no strong evidence of a difference between opt‐in and opt‐out countries.^2^

In 1997 Brazil introduced an opt‐out law.^25^ Despite not being required, in practice almost all surgeons asked for family approval before removing organs. As a result, the government added a new paragraph to the law, stating that doctors should get permission from relatives before organ retrieval. However, part of the Brazilian population feared that their organs would be removed even before they were clinically dead, and many responded by registering as nondonors. As a result, in 1998 the law was abolished and Brazil returned to an opt‐in law.^26^ In contrast, Spain introduced an opt‐out system in 1979^27^, ^28^ and is an international leader in rates of deceased donation.^29^ However, Spain operates a ‘soft opt‐out’ system, and rather than the high donation rates being attributed to the law, Spanish transplant professionals have attributed this to organisational interventions to optimize transplant and organ retrieval work forces, education of healthcare professionals, early and sensitive family approach and guidance to reduce inappropriate discarding of suitable organs.^30^

Some Spanish transplant professionals believe national media campaigns have contributed to the high deceased organ donation rates in Spain.^30^ Media campaigns are underway in the United Kingdom to raise awareness of the law change: These focus on making family members aware of one's own wishes regarding donation, to assist decision‐making by families at the time of death. Patients in our study suggested that rather than focusing on the donor and donation, campaigns should highlight the positive impact of organ donation on transplant recipients. These ‘gain‐framed’ messages have previously been shown to generate favourable reactions regarding organ donation,^31^, ^32^, ^33^ but messages based on reciprocity (e.g., ‘If you needed an organ transplant would you have one? If so please help others’).^33^, ^34^ appear even more effective.

Expectations of an increase in deceased organ transplants may impact on behaviours of clinicians, patients and family members/potential donors regarding living organ donation. Living‐donor transplant rates in Spain are low (7.2 pmp in 2019) and fall behind those of Australia (9.4 pmp), Norway (12.5 pmp), Canada (15.0 pmp), the United Kingdom (15.7 pmp) and the Netherlands (30.3 pmp).^35^ Previous small surveys have found that people on the waiting list in Spain do not hold favourable views towards living‐donor transplantation^36^: In a questionnaire study of those on the kidney transplant waiting list, only 35% would accept a related living kidney if it were offered to them, with 60% preferring to wait for a deceased‐donor organ.^37^ This was not due to a lack of offered organs as 66% of participants reported that a family member/friend had offered to donate a kidney to them. Although not explicitly investigated in these Spanish studies, the reluctance to accept a living‐donor transplant may be affected by a patient's reasonable expectations of receiving a deceased‐donor transplant within a relatively short waiting time.^27^ In our study we found that patients had high expectations of increased deceased‐donor numbers as a result of the opt‐out law and therefore a similar reluctance to accept a living‐donor transplant may be observed in the United Kingdom.

Healthcare professional participants described how they would continue to discuss the option of a living‐donor transplant. Clinicians should ensure patient expectations are realistic so that a decision to reject an offered living‐donor transplant is not based on an expectation of an increased likelihood of a deceased‐donor transplant, which is not evidence‐based. All healthcare professionals should aim to discuss with patients the current evidence regarding deceased‐donor organ availability, to ensure accurate information informs decision‐making. The UK Renal Registry has recently introduced a patient summary of their annual report^38^ and NHSBT has produced summary infographics to accompany their Organ Specific Reports, which may help to ensure that the information on donation and transplantation rates is more accessible to patients.

To our knowledge, this is the first qualitative study to investigate the expectations of a move to opt‐out legislation in the United Kingdom. There are some limitations: (i) Although a diverse sample of patients and healthcare workers was achieved, and theme saturation for this mixed stakeholder group reached, 36% of those invited agreed to participate, and only four family members were interviewed. Recruitment was likely affected by the COVID‐19 pandemic, which coincided with this study. In particular, the COVID‐19 pandemic meant that family members were not allowed to attend hospital appointments with patients and therefore exposure to waiting room posters was limited. (ii) The study population was limited to individuals with kidney disease, their family members or healthcare professionals who work with them. Individuals eligible for other solid organ transplants were not invited to participate. The vast majority of organ transplants are kidney transplants: More than 75% of those on the transplant waiting list are awaiting kidney transplantation^1^ but findings may not be transferable to candidates for other solid organ transplants and those who work with them. (iii) Interviews were carried out by a clinician (P. K. B.), known to the healthcare workers. Although participants spoke freely we are unable to determine if this altered responses.

## 5 CONCLUSIONS

We have described the expectations of people with kidney disease, family members and healthcare practitioners with respect to the change to opt‐out organ donation legislation in England. Our findings suggest that patients' and clinicians' expectations of the impact of the legislation differ. The ‘hopeful’ expectations of patients are not currently supported by emergent evidence. Clinicians suggested that they would continue to promote living‐donor transplantation, partly due to low expectations regarding the positive impact of the law change.

## CONFLICT OF INTERESTS

The authors declare that there are no conflict of interests.

## AUTHOR CONTRIBUTIONS

Pippa K. Bailey, Fergus J. Caskey and Yoav Ben‐Shlomo participated in research design; Pippa K. Bailey, Hannah Lyons, Fergus J. Caskey, Yoav Ben‐Shlomo, Mohammed Al‐Talib and Lucy E. Selman participated in the writing of the paper; Pippa K. Bailey and Adarsh Babu participated in the performance of the research; Pippa K. Bailey, Hannah Lyons and Mohammed Al‐Talib participated in data analysis.

## Supporting information

Supporting information.

Supporting information.

## Data Availability

This study generated qualitative data in the form of digital audio recordings and transcripts from interviews. Participants were asked to provide written consent to share their anonymised data with other researchers. Data will be shared only if consent has been provided. Twenty‐nine of Thirty‐two participants provided consent for data sharing. Anonymised interview transcripts have been uploaded to the University of Bristol's Research Data Repository: https://data.bris.ac.uk/data/. Audio files of the recorded interviews are not suitable for sharing as they carry a high risk of allowing the research participant to be identified, and the content of interviews includes sensitive information. Individuals who wish to access the data set can contact the researchers directly or actively search the University of Bristol's data repository. Although the qualitative transcripts have been anonymised, as personal and sensitive issues have been discussed we cannot rule out the risk of identification, and therefore access to these transcripts is controlled. Individual researchers will need to request access to the controlled data through the University of Bristol via the Data Access Committee (DAC) for approval before data can be shared after their host institution has signed a DAC. The procedure for accessing data can be found here: https://www.bristol.ac.uk/staff/researchers/data/accessing-research-data/.
