# Supplementary material for: Expectations of a new opt‐out system of consent for deceased organ donation in England: A qualitative interview study
Source: Health Expect. 2021 Dec 24;25(2):607–16. doi: 10.1111/hex.13394 (PMC8957744; doi:10.1111/hex.13394)
Supplement: Supplementary file 2 — Supporting information. [file HEX-25--s002.pdf]

**Supplementary Material – Example topic guide – for patient participant interviews**

| Topic | Questions |
|---|---|
| Communication on LDKT | Have you talked to your family and close friends about the possibility of having a kidney transplant? Was/Is this difficult? If yes, what makes it difficult? Did anything make it easier? |
| | Did you ever directly ask somebody to give you a kidney? What happened? |
| Attitudes towards obtaining and providing information | Did/Do you feel you have enough information on kidney transplantations and kidney donation? |
| | Did/Do you feel happy you understood/understand everything? |
| | Are you happy to ask your doctor or kidney nurse questions if you have them? Is this easy to do? |
| Norwegian approach | In Norway the kidney doctor asks the person with kidney disease about who is in their family and any close friends. If the patient agrees, the doctor then contacts these family members and friends asking them to think about donating a kidney. They are invited to come to the hospital to talk about kidney donation.<br>What do you think of this approach? |
| | Does anything appeal to you about this? Does anything worry you about this? |
| | Discuss wording/content of letter and living donation information sheet. Review drafted resources. |
| Home-based educational intervention | In the Netherlands and in parts of America, nurses or psychologists visit people with kidney disease and their families at their homes. People are encouraged to invite any family members and friends who don't live at home to come to the meeting. The nurses/psychologists talk to everyone about kidney disease, transplants and kidney donation, they help to start conversations about possible kidney donation, and can answer any questions people have face to face. They can visit once or twice, and leave information sheets, DVDs, website links etc.<br>What do you think of this approach? |
| | What would you like to discuss during such a meeting? |
| | Do you think that people in your family would be open to this? |
| | Can you think of people who might not want something like this? |
| | Do you think other places (e.g. café, church) would be alternatives to going into someone's home? |
| | The nurses and psychologists usually take information sheets and can provide DVDs and weblinks. Do you think this is helpful? |
| | Discuss animation content. |
| Transplant Candidate Advocates (TCAs) | People tell us it can be very difficult to talk to family and friends about possible kidney donation.<br>In some parts of America, doctors ask people with kidney disease to pick a family member or friend who receives training on living-donor kidney transplantation, and in how to start conversations about kidney donation. This person is given information leaflets and is trained as an advocate: someone willing to speak to other friends/family about donation on the patient's behalf. They can also go to clinic appointments with the patient and ask questions and find out information.<br>What do you think of this approach? |
| | Can you think of anyone you might ask to do this? |
| | Do you think that people in your community would be open for this intervention? |
| Other suggestions | Is there another approach you think might be helpful? |

| Topic | Questions |
|---|---|
| Opt-out legislation | In May 2020 England will move to an ‘opt-out’ law for organ donation after death. Were you aware of this? What do you think about this? |
| | What impact do you think the law change will have? How do you think the change in the law will affect you? Probe if participant expects law change to increase number of organs available for transplant. |
| | Will the change in the law affect any of the decisions you’ve made about transplants? Probe as to whether it would change willingness to accept a living donor kidney transplant. |
| | Do you think the change in law should be highlighted in a media campaign, for example on TV? Investigate participant views on content of adverts. |
| | Do you think a media campaign should focus on donation after death or should it include living donation? |

**Example topic guide – for healthcare practitioners**

| Topic | Questions |
|---|---|
| Views on helping people to find a donor | Do you think that healthcare professionals have a role in helping people with kidney disease to find a living kidney donor?<br><br>What do you think that role is? |
| | Do you think there are any limits to what healthcare workers should do to help someone to find a kidney donor?<br><br>Are there any things you think are unacceptable? |
| Experience of ever asking a family member directly to donate | Have you ever asked a patient's family members directly whether they would consider donating? How did you do this? (including e.g. in person, over the phone, by letter) |
| | How do you feel about asking family members to consider donation? |
| Norwegian approach | In Norway the kidney doctor asks the person with kidney disease about who is in their family and about any close friends. If the patient agrees, the doctor then telephones or writes to these family members and friends asking them to think about donating a kidney. They are invited to come to the hospital to talk about kidney donation.<br><br>What do you think of this approach?<br><br>Does anything appeal to you about this? Does anything worry you about this? |
| | Do you think that your NHS trust and colleagues would be supportive of this approach? |
| | Who do you think the letter should come from? e.g. Renal unit, named consultant, living donor team |
| Home-based educational intervention | In the Netherlands and in parts of America, health care workers arrange to visit people with kidney disease and their families at their homes. The healthcare workers talk to everyone about transplants and kidney donation, help to start conversations about possible kidney donation, and can answer any questions people have face to face. They can visit once or twice, and leave information sheets, DVDs, website links etc.<br><br>What do you think of this approach? |
| | What do you think it would be important to cover during such a meeting? |
| | Who do you think should do the home visits? |
| | Can you think of people who might not want something like this? |
| | Do you think other places (e.g. café, church) would be alternatives to going into someone's home? |
| | Do you think that your NHS trust and colleagues would be supportive of this approach? |
| Transplant Candidate Advocates (TCAs) | A lot of people tell us it can be very difficult to talk to family and friends about possible kidney donation. In some parts of America, doctors ask people with kidney disease to nominate a family member or friend who receives training on living donor kidney transplantation, and in how to start conversations about kidney donation. This person is given information leaflets and is trained as an advocate: someone willing to speak to other friends/family about donation on the patient's behalf. They can also go to clinic appointments with the patient and ask questions and find out information.<br><br>What do you think of this approach? |
| | Do you think that your NHS trust and colleagues would be supportive of this approach? |
| Other suggestions | Is there another approach you think might be helpful? |

| Topic | Questions |
| --- | --- |
| **Opt-out legislation** | **In May 2020 England will move to an 'opt-out' law for organ donation after death. Were you aware of this? What do you think about this?** |
| | **What impact do you think the law change will have?**<br>**Probe what impact is expected on: deceased organ donation/deceased donor numbers, and living donors. Probe if participant expects law change to increase number of organs available for transplant overall. Probe sources of information for these views.** |
| | **Will you change your practice because of the law change?**<br>**Probe whether the participant's expectations of the impact of the law change will impact practice. Explore existing recommendations to patients regarding deceased versus living donor transplants. Will those who have said that they expect an increase in deceased donor transplants be less likely to recommend/support patients to pursue living donor transplants?** |
| | **Do you think the change in law should be highlighted in a media campaign, for example on TV? Investigate participant views on content of adverts.** |
| | **Do you think a media campaign should focus on donation after death or should it include living donation?** |

**Example topic guide – for family and friends**

| Topic | Questions |
|---|---|
| Communication on LDKT | Has your family member with kidney disease ever talked to you and the rest of your family about the possibility of having a kidney transplant? How did you find this? Was it difficult? If yes, what makes it difficult? Did anything make it easier?<br>Who started these conversations? |
| | Before this interview did you know about the possibility of living kidney donation?<br>Have you or anyone else in your family ever offered to give your family member a kidney?<br>If yes, what happened?<br>If no, can you tell me more about why? |
| Attitudes towards obtaining and providing information | Did/Do you have enough information on kidney transplantations and kidney donation? |
| | Did you feel happy you understood everything? |
| | Did/do you have an opportunity to ask a doctor or nurse any questions you had? |
| Norwegian approach | In Norway the kidney doctor asks the person with kidney disease about who is in their family and about any close friends. If the patient agrees, the doctor then telephones or writes to these family members and friends asking them to think about donating a kidney. They are invited to come to the hospital to talk about kidney donation.<br>What do you think of this approach? |
| | Does anything appeal to you about this? Does anything worry you about this? |
| Home-based educational intervention | In the Netherlands and in parts of America, nurses or psychologists visit people with kidney disease and their families at their homes. People are encouraged to invite any family members and friends who don't live at home to come to the meeting. The nurses talk to everyone about kidney disease, transplants and kidney donation, they help to start conversations about possible kidney donation, and can answer any questions people have face to face. They can visit once or twice, and leave information sheets, DVDs, website links etc.<br>What do you think of this approach? |
| | What would you like to discuss during such a meeting? |
| | Do you think that other people in your family would be open to this? |
| | Can you think of people who might not want something like this? |
| | Do you think other places (e.g. café, church) would be alternatives to going into someone's home? |
| | The nurses and psychologists usually take information sheets and can provide DVDs and weblinks. Do you think these are helpful? |
| Transplant Candidate Advocates (TCAs) | People tell us it can be very difficult to talk to family and friends about possible kidney donation. In some parts of America, doctors ask people with kidney disease to nominate a family member or friend who receives training on living donor kidney transplantation, and in how to start conversations about kidney donation. This person is given information leaflets and is trained as an advocate: someone willing to speak to other friends/family about donation on the patient's behalf. They can also go to clinic appointments with the patient and ask questions and find out information. What do you think of this approach? |
| | Is this something you would be willing to do? |
| | Do you think that people in your community would be open for this intervention? |
| Other suggestions | Is there another approach you think might be helpful? |

| Topic | Questions |
|---|---|
| Opt-out legislation | In May 2020 England will move to an 'opt-out' law for organ donation after death. Were you aware of this? What do you think about this? |
| | What impact do you think the law change will have? How do you think the change in the law will affect you? Probe if participant expects law change to increase number of organs available for transplant. |
| | If participant is a kidney donor, probe whether they think the law change would have affected their decision to donate? Explore participant's attitude towards living kidney donation. Investigate whether the law change will change this attitude. |
| | Do you think the change in law should be highlighted in a media campaign, for example on TV? Investigate participant views on content of adverts. |
| | Do you think a media campaign should focus on donation after death or should it include living donation? |
